# The Mental Health of People Living with HIV in China, 1998–2014: A Systematic Review

**DOI:** 10.1371/journal.pone.0153489

**Published:** 2016-04-15

**Authors:** Lu Niu, Dan Luo, Ying Liu, Vincent M. B. Silenzio, Shuiyuan Xiao

**Affiliations:** 1 Department of Social Medicine and Health Management, School of Public Health, Central South University, Changsha, China; 2 Department of Psychiatry, University of Rochester Medical Center, Rochester, New York, United States of America; University of Missouri-Kansas City, UNITED STATES

## Abstract

**Background:**

Understanding the mental health burdens faced by people living with HIV in China is instrumental in the development of successful targeted programs for psychological support and care.

**Methods:**

Using multiple Chinese and English literature databases, we conducted a systematic review of observational research (cross-sectional, case-control, or cohort) published between 1998 and 2014 on the mental health of people living with HIV in China.

**Results:**

We identified a total of 94 eligible articles. A broad range of instruments were used across studies. Depression was the most widely studied problem; the majority of studies reported prevalence greater than 60% across research settings, with indications of a higher prevalence among women than men. Rates of anxiety tended to be greater than 40%. Findings regarding the rates of suicidality, HIV-related neurocognitive disorders, and substance use were less and varied. Only one study investigated posttraumatic stress disorder and reported a prevalence of 46.2%. Conflicting results about health and treatment related factors of mental health were found across studies.

**Conclusions:**

Despite limitations, this review confirmed that people living with HIV are vulnerable to mental health problems, and there is substantial need for mental health services among this population.

## Introduction

Although the nationwide HIV prevalence remains low in China, the number of people living with HIV, as well as the number of new infections per year, continues to increase [1]. At the end of October 2014, over 0.49 million people were living with HIV in China based on the China information system for disease control and prevention, of which 40% were AIDS patients [2]. Case reporting data shows that from 2011 to 2014, the number of newly diagnosed increased each year, with the figures for each year standing at 20,450, 41,929, 42,286, and 45,145, respectively [3,4]. The healthcare and management of such a vast number of people living with HIV is a realistic challenge for Chinese health service providers and policy makers, particularly because this number continues to grow.

Mental health in relation to people living with HIV is becoming an increasing concern worldwide; however, to date this pressing issue has been largely ignored in global policy guidelines [5]. Since 2003, under the “Four Frees, One Care” policy, it has become easier for people living with HIV in China to access HIV-related health care [6], but their psychiatric and psychological needs are yet seldom touched [7]. HIV infection is regarded as a traumatic and stressful experience that can negatively affect mental health status and potentially lead patients into a cycle of physical and mental decline [8]. Studies have shown that people living with HIV are more likely than the general population to exhibit mental health problems including depression, anxiety, and suicidality, as well as the harmful use of substances [8–11]. The chronic effects of HIV and antiretroviral therapy (ART) on the brain can also result in HIV-associated neurocognitive disorders (HAND) [12]. Poor mental health status can serve as a barrier to adequate ART adherence, and consequently decrease quality of life and increase mortality [8–10]. Policies and programs designed to decrease the mental health burdens of people living with HIV are urgently needed, and appropriate response hinges on systematic information about mental health status.

In this systematic review, we integrated data on the mental health problems of Chinese people living with HIV from articles published from 1998 to 2014. We sought to accomplish four aims. First, we reviewed articles for evidence of disparities in mental health morbidity affecting people living with HIV. Second, we identified the health and treatment related correlates of mental health in this population. Third, we identified some of the methodological issues that currently influence our understanding of mental health morbidity concerns among this population. Finally, we provided our thoughts on important future directions for research on the mental health of people living with HIV in China.

## Methods

### Search strategy

We followed the Preferred Reporting Items for Systematic Reviews and Meta-Analyses: the PRISMA guideline [13,14]. We systematically searched the following databases: (1) international databases including MEDLINE, PsychINFO, PubMed, and Web of Science; and (2) Chinese scientific databases including China National Knowledge Infrastructure Project (CNKI), China Biomedical Literature Database (CBM), and Digital Journal of Wan fang Data (Wan fang).

We conducted our search by combining keywords from the following concepts:

1. The population: HIV, AIDS;
2. The outcome: mental health, psychology, depression, anxiety, substance use, alcohol use, drug use, smoking behavior, suicide, post-traumatic stress disorder, and neuropsychology;
3. The study location: China.

The full search strategy with adapted terms for each database is included in S1 File.

### Eligibility criteria

We reviewed abstracts and full texts of retrieved articles according to the following inclusion criteria: (1) studies conducted in mainland China or Hong Kong with people living with HIV; (2) written in English or Chinese; (3) an observational design (cross-sectional, case-control, or cohort); and (4) contains quantitative data on mental health (prevalence, or scores of instruments).

Articles were excluded if they were qualitative studies, literature reviews, conference abstracts, pharmacological studies, or intervention evaluations. In terms of duplicated data, the one with the maximum sample size and the most comprehensive results was included.

### Data

We extracted data on study population (age and gender distribution, HIV-related clinical information), study site, study design, sampling method, instruments, prevalence of mental health problems, and the health and treatment-related correlates.

Because we focused on sample characteristics and prevalence of mental health problems for this review, a four-item appraisal checklist adapted from the scoring systems developed by Loney et al [15] and Kim et al [16] was used for each article. The checklist evaluated sample size (≥ 300 vs. < 300), sampling method (random vs. convenient), participation rate (reported vs. unreported or < 70%), and eligibility criteria (provided vs. not provided). Total scores ranged from 0 to 4, with a lower number indicating lower quality and a higher risk of bias.

## Results

### Search and study selection

**Fig 1:**
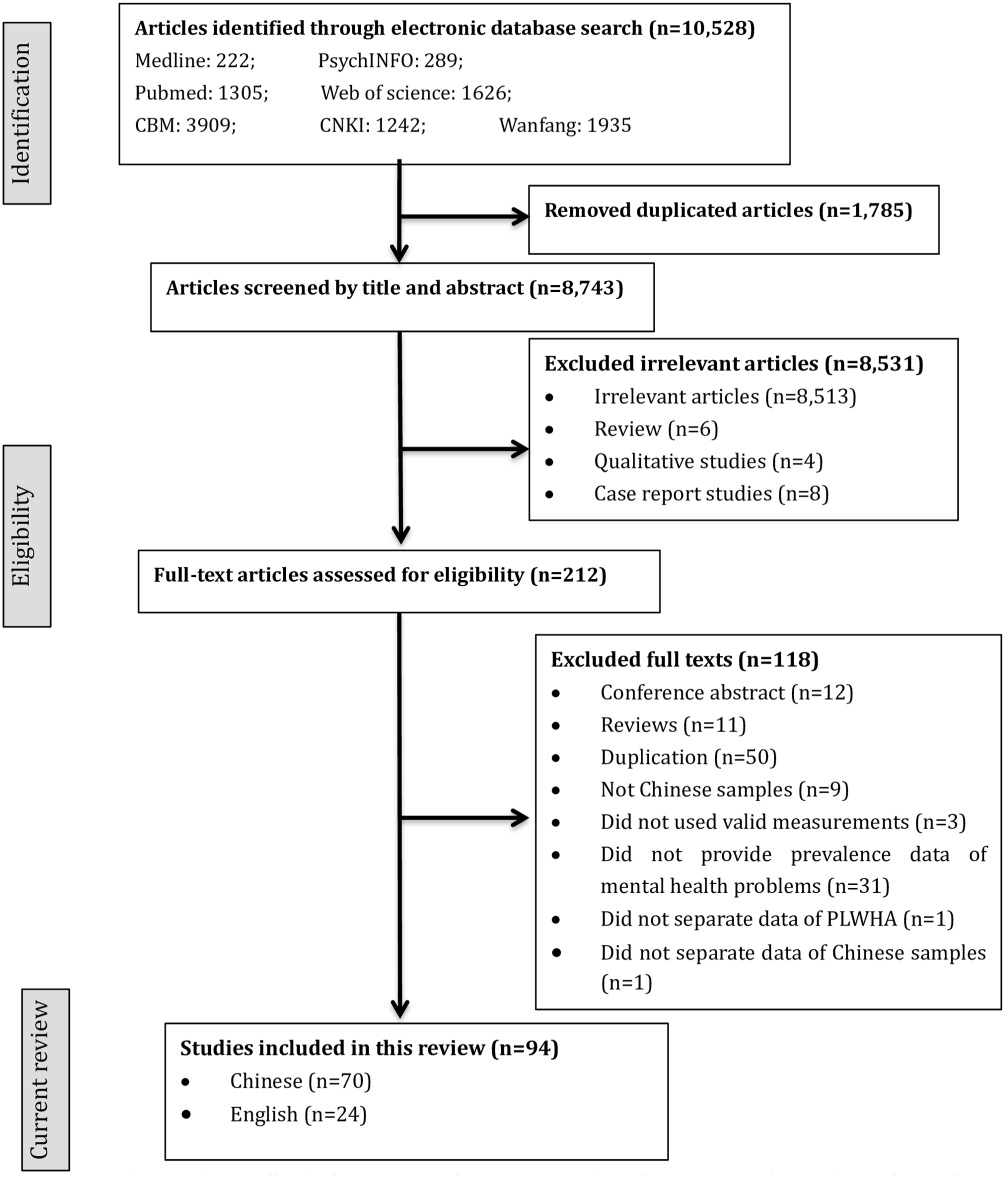
PRISMA flowchart of study selection for systematic review of published research on prevalence of mental health problems among people living with HIV.

Our electronic search yielded 10,528 articles: 1,784 duplicate articles were retrieved from more than one database and 8,532 irrelevant articles were excluded. We assessed 212 full-text articles for eligibility. Ninety-one studies reported in 94 articles (70 Chinese and 24 English articles published between 2004 and 2014) were included in this systematic review, because one study reported findings across two articles [17,18] and another across three articles [19–21]. Fig 1 presents the flowchart of our study selection and the frequency of reasons for exclusion.

### Characteristics of studies

Seventy-six out of 91 studies provided province-specific data; seven were conducted in multiple provinces and five did not report the study region. One third (n = 31) of the studies did not specify participants’ stage of illness or the time since being diagnosed HIV-positive. Several studies focused on specific sub-populations. All community-based studies sourced participants from rural areas [19–33] predominantly in central China where most participants were former blood/plasma donors [19–27]. There were also three other studies about people with combined HIV and tuberculosis (HIV/TB) [34–36], three about people who injected drugs [35,37,38], three about pregnant women [31,39,40], and three about men who have sex with men [41–43].

Regarding the risk of bias of individual studies, over half of the studies (54.9%, 50/91) had a score of 0 or 1, which indicates a high risk of bias. Sample sizes ranged from 16 to 1,064, and 74 studies (81.3%) were with a sample size less than 300. Forty-one studies (45.1%) did not provide eligibility criteria, and 53 (58.2%) did not report participation rate. The most common limitation observed was risk of bias attributable to convenience sampling (29.7%, 27/91), while 35 studies (38.5%) did not specify their sampling method (Table 1). Only 15 studies used random sampling, and 22 included HIV-negative comparison groups. The overview of studies is provided in S1 Table.

**Table 1:** Characteristics of reviewed articles.

	Studies, No.	%(N = 91)
**Sub-population**		
Former blood/plasma donors	9	9.9
HIV/TB	3	3.3
Injected drug users	3	3.3
Pregnant women	3	3.3
Men who have sex with man	3	3.3
Unspecific	70	76.9
**Study type**		
Cross-sectional	65	71.4
Case-control	22	24.1
Cohort	2	2.2
Other	3	3.3
**Sample method**		
Convenience	27	29.7
Random	17	18.7
Complete	9	9.9
Other	6	6.6
Not available	32	35.1
**Sample size**^a^		
≤50	11	12.1
51–100	21	23.1
101–200	27	29.6
201–300	16	17.6
>300	16	17.6
**Research topic**		
Depression	78	85.7
Anxiety	64	70.3
Suicide	11	12.1
PTSD^b^	1	1.1
HAND^c^	9	9.9
Substance use	18	19.8

^a^Sample size of people living with HIV

^b^Post-traumatic stress disorders

^c^HIV-associated neurocognitive disorders

### Mental health problems

#### Depression

Three studies reported rates of clinical disorder by using diagnostic interviews, i.e., the Composite International Diagnostic Interview (CIDI, Chinese version) [21,38] and the Structured Clinical Interview for DSM-IV (SCID-I/P, Chinese version) [44], among different samples from different settings. In a CDC-based study, Ren and colleagues [44] found 22.8% had lifetime major depressive disorder in a cross-sectional convenience sample of 342 adults who were HIV-positive in Guangdong (14.8% female; 79.5% were drug users). In their community-based study with a convenience sample of 203 rural HIV-positive former plasma donors (39% female), Atkinson and colleagues [21] found 14% had lifetime major depressive disorder and 2.0% had a current major depressive episode. Participants that were HIV-positive were more likely to experience lifetime major depressive disorder than HIV-negative controls (5%). Jin et al. [38] found that 6.4% of a convenience sample of 204 HIV-positive persons who used heroin in methadone clinics (34.2% female) had experienced major depression in their lifetime, while 1.0% were having a current major depressive episode. The rates did not differ meaningfully between the HIV-positive and HIV-negative people who used drugs in treatment. Compared with the control group who were HIV-negative and did not use drugs (1.5%), the rate of lifetime major depression in people who used heroin was significantly higher.

In addition, 78 studies assessed depressive symptoms using a broad range of instruments (Chinese versions): 27 studies used the Symptom Check List-90 (SCL-90) [23,26,33,34,36,40,45–65]; 26 used the Zung Self-Rating Depression Scale (SDS) [17,18,29–32,34,35,51,57,63,65–80]; seven used the Beck Depression Inventory (BDI) [19–21,25,38,81–84]; six used the Centers for Epidemiological Studies Depression Scale (CES-D) [85–90]; three used the Hamilton Depression Scale (HAMD) [37,47,91,92]; three used the Depression Anxiety Stress Scale (DASS) [22,24,41]; three used the Hospital Anxiety and Depression Scale (HADS) [91,93,94]; two used the Patient Health Questionnaire Depression Scale (PHQ-9) [95,96]; and one used the Irritability, Depression and Anxiety Scale (IDA) [97].

The median prevalence of depressive symptoms among people living with HIV was 60.64%, with a range of 16% to 100%. Twelve studies compared the prevalence of depression among men and women, and women (36.6%–94.5%) were more likely to report depression than men (37.9%–71.8%) [22,46,48,50,56,67,70,75,79,82,90,94]. In addition, seventeen studies documented higher prevalence of depression and more severe depressive symptoms among persons who were HIV-positive when compared to HIV-negative controls [24,26,30,34,36,40,45,48,52,55,56,59,66,76,92,93,97].

#### Anxiety

Only one study presented prevalence of clinical diagnosis, which was a CDC-based study conducted in Guangdong province. Using the SCID-I/P, it was found that 15.8% of the participants met the criteria for lifetime general anxiety disorder [44].

However, anxiety symptoms were prevalent in a number of studies. Sixty-four studies assessed anxiety symptoms mainly though measurement instruments (Chinese versions): 27 studies used the SCL-90 [23,26,33,34,36,40,45–65]; 26 used the Zung Self-Rating Anxiety Scale (SAS) [25,29–31,35,42,51,57,63,65–68,70,71,74,76,78,80,86,90,98–102]; three used the DASS [22,24,41]; three used the HADS [91,93,94]; two used the Hamilton Anxiety Scale (HAMA) [47,92]; two used the General Anxiety Disorder-7 (GAD-7) [95,96]; and one used the IDA scale [97].

The prevalence of anxiety symptoms ranged from 11.11% to 97.53%, and the median prevalence was 43.13%. Eight studies reported that women (47%–80%) were also more likely to report anxiety than men (41.3%–58.6%)[46,48,50,56,70,90,94,102]. Additionally, seventeen studies compared prevalence of anxiety between people living with HIV and HIV-negative controls, which showed that people living with HIV were more likely to experience anxiety and have more severe anxiety than people who were HIV-negative [24,26,30,34,36,40,45,48,52,55,56,59,76,92,93,97,98].

#### Suicidal behavior

Two studies reported death records due to suicide [103,104]. Qu and colleagues [103] reviewed medical records from Beijing Di Tan Hospital (1991–2003) and found that among 848 HIV-positive patients, nineteen (2.2%) died from suicide (5 females) and four had made suicide attempts (1 female). Seventeen patients were farmers and nine died by poison. Lai et al. [104] collected data from the DataFax Antiretroviral Therapy Information System in China between July 2003 and September 2009. There were 766 people living with HIV who started ART before October 2008 in Sichuan province, and three died from suicide among 144 death records (2.1%) [104].

Nine studies examined suicidal behavior (suicidal ideation, plan, and attempt), but definitions were highly variable and most failed to present gender-disaggregated data (Table 2). Several studies investigated suicidal ideation and suicide attempt in participants’ lifetimes [28,44], in the past year [22,27,44], in the past six months [26], or since HIV diagnosis [41], by asking simple questions like “Have you thought about suicide/attempted suicide?” One study measured suicidal ideation by using the Self-Rating Idea of Suicide Scale (SIOSS), in which a high score indicates a high level of suicidal ideation [18]. Two studies assessed lifetime suicidality and current suicidality by using the CIDI and BDI respectively [21,38].

**Table 2:** Summary findings of suicide behaviors.

Form of suicidality	Measures	First author, year	Prevalence
Completed suicide	Death records	Qu, 2005	19/848 (2.2%)
		Lai, 2011	3/766 (2.1%)
Suicide attempts	Medical records	Qu, 2005	4/848 (0.5%)
	Single item	Lv, 2007	5.9% (past 1 year)
		Lau, 2010	8% (past 1 year)
		Wu, 2014	2.67% (since HIV diagnosis)
	Questionnaire	Wu, 2007	6.9% (lifetime; male 6.5%; female 7.1%)
		Ren, 2009	37.7% in lifetime; 29.5% in the past year;
		Su, 2010	0.7% (past 6 months)
	CIDI (3.0)	Atkinson, 2011	HIV+: 2%; HIV-: 1% (lifetime)
Suicide ideation	Single item	Lv, 2007	32.3% (past 1 year; 58.5% were female)
		Lau, 2010	34.1% (past 1 year)
		Wu, 2014	48% (since HIV diagnosis)
	Questionnaire	Wu, 2007	34.8% (lifetime; male: 24.7%; female: 23.5%)
		Ren, 2009	13.7% in lifetime; 3.8% in the past year
		Su, 2010	5.9% (past 6 months)
	SIOSS	Qin, 2014	29.14% (SIOSS≥12; male: 24.74%; female: 38.30%)
	CIDI (3.0)	Atkinson, 2011	Think a lot about death: HIV+ 16%; HIV- 7%. Think about suicide: HIV+ 11%; HIV- 6%.(in lifetime)
	BDI (Item 9)	Atkinson, 2011	HIV+ 14%; HIV- 12% (past 2 weeks)
		Jin, 2013	HIV+IDU: 37.1%; HIV-IDU: 43.2%; Non-IDU: 8.5% (past 2 weeks)
Suicide plan	Questionnaire	Wu, 2007	25/178 (14.0%; lifetime)
		Su, 2010	2.6% (past 6 months)
	CIDI (3.0)	Atkinson, 2011	HIV+ 8%; HIV- 3% (lifetime)

Notes: CIDI = Composite International Diagnostic Interview; BDI = Beck Depression Inventor; SIOSS = Self-rating Idea of Suicide Scale; Questionnaire = not specified or self-developed instruments.

A few noteworthy findings emerged from these studies about suicidal behavior. The prevalence of suicidal ideation in the past year ranged from 29.5% to 34.1% [22,27,44], and the prevalence of attempted suicide in the past year varied from 3.8% to 8% [22,27,44]. There was no significant gender difference observed [27,44]. Additionally, in one community-based study, twelve rural participants (6.9%) had attempted suicide in their lifetime and mostly after getting an HIV diagnosis (10/12) [28]. In a convenience sample of 225 newly diagnosed men who have sex with men in Chengdu, Wu et al. [41] found almost half (48%) of them reported suicidal ideation and 2.67% had attempted suicide since diagnosis. In case-control studies, people who were HIV-positive appeared to be more likely to report suicidal behaviors than their HIV-negative counterparts [21,26,56].

#### Posttraumatic stress disorder (PTSD)

We found only one study that screened PTSD symptoms among people living with HIV in China [105]. This survey was conducted in Hengyang, Hunan in 2013 with information collected from a convenience sample of 264 participants aged 18 to 74. Over half of the participants were in the HIV stage (64.4%) and on ART (66.3%). The Chinese version of the PTSD Checklist (PCL) was used and the prevalence of PTSD was 46.2% (male 39.4%; female 59.6%).

#### HIV-associated neurocognitive disorders (HAND)

Wu et al. [69] reviewed clinical records of 36 AIDS patients treated from 1999 to 2003 in two hospitals in Shanghai. There were six patients (16.7%) with confirmed AIDS dementia complex (ADC). The average survival time of these ADC patients since diagnosis was 4.7 months, and the average age at death was 41.8 years [69].

As shown in Table 3, six studies examined neuropsychological (NP) impairment or HAND using a wide range of measurements: four studies cited different NP test batteries [19,20,85,88,106], two studies cited the International HIV Dementia Scale (IHDS) [106,107], and one cited the Montreal Cognitive Assessment (MoCA) [108].

**Table 3:** Summary findings of HIV-associated neurocognitive disorders.

Measurement tool	First author, year	Prevalence
Medical records	Wu Y, 2007	6/36 (16.7%) with confirmed ADC
Neuropsychological test battery	Heaton, 2008; Cysique, 2010	**Baseline:** HIV+: 36.8% (HIV-monoinfected: 34.2%; HIV/HCV coinfected: 39.7%); HIV-: 19.3% (HCV-monoinfected: 37.2%; controls: 12.7%); **1-year follow-up** (NP decline): HIV+: 27.6%; HIV-: 5%.
	Wright, 2008	4% in Beijing; 23% in Hong Kong
	Zhang, 2012	50/134 (37.31%)
	Dwyer, 2014	69.4%
International HIV Dementia Scale (IHDS)	Zhang, 2012	52/134 = 38.1% (ANI: 22.4%; MND: 11.9%; HAD: 4.5%)
	Zhao, 2013	37.4(ANI: 18.2%; MND: 10.9%; HAD: 8.3%)
Montreal Cognitive Assessment (MoCA)	Zhen, 2013	52.2% (MoCA≥26)
Hong Kong List Learning Test (HKLLT)	Au, 2008	(Mild memory impairment) Total learning: 18%; 10-min Delay Recall: 28%; 30-min Delay Recall: 29%; Discriminability: 13%.

Notes: ADC: AIDS dementia complex; NP decline: Neuropsychological decline; ANI: asymptomatic neurocognitive impairment; MND: neurocognitive disorder; HAD: HIV-associated dementia

Among these studies, 4% to 69% of the participants were classified as being neuropsychologically impaired [19,20,85,88,106]. Notably, in a community-based cohort study [19,20], 203 HIV-positive people and 198 HIV-negative controls were selected from a rural county in Central China. At baseline, NP impairment was found in 34.2% of the HIV-monoinfected group and 39.7% of the HIV/HCV co-infected group, compared to 37.2% of the HCV-monoinfected group and 12.7% of the uninfected controls. HIV-positive participants with AIDS were more likely to be impaired (43%) than non-AIDS patients (29%) [19]. Twelve months later, 192 HIV-positive and 101 HIV-negative participants were reassessed, and 27% of HIV-positive individuals developed significant cognitive decline compared with 5% of HIV-negative individuals [20].

In addition, Au et al. [81] assessed memory deficits among 90 HIV-positive individuals in Hong Kong using the Hong Kong List Learning Test, and 13.3% to 28.9% were found to have mild memory impairment in the different functions measured.

#### Substance use

Findings related to substance use were much more fragmented, and inconsistent measurement made results across studies hard to interpret (Table 4). Only two studies defined alcohol use disorder and other substance use disorders through the CIDI (3.0) [21,56]. Atkinson et al. [21] observed a 14% prevalence of lifetime alcohol use disorder in 203 HIV-positive rural former plasma donors compared to 6% lifetime prevalence in a comparable sample of 198 HIV-negative rural former plasma donors. All diagnoses of a lifetime alcohol use disorder were in men and no other substance use disorder was found. Jin and colleagues [56] found comparable rates of lifetime alcohol use disorder among HIV-positive people who used drugs, HIV-negative people who used drugs, and the control group, which were 15.7%, 19.3%, and 12.4%, respectively. The prevalence of current (last 30 days) alcohol use disorders was 2% in all three groups. In addition, 1% of HIV-positive people who used drugs and 3% of HIV-negative people who used drugs had current heroin use disorders.

**Table 4:** Summary findings of substance use.

Form of substance use	Measures	First author, year	Prevalence
Substance use	Questionnaire	Li, 2004	HIV+: 2.01±1.35; Relatives: 0.62±0.83; Control: 0.67±0.79 (definition unknown)
Drug use	CIDI (3.0)	Atkinson, 2011	None
		Jin, 2013	Current heroin use disorders: HIV+IDU: 1.0%; HIV-IDU: 3.0%
	Questionnaire	Fang, 2008	4.74% (definition unknown)
		Shan, 2009	98.4% (lifetime)
		Ren, 2009	79.5% (lifetime)
		Greene, 2013	16.7% (daily)
		Luo, 2013	55.4% (lifetime)
		Wang, 2014	23.2% (current heroin use)
Alcohol use	CIDI (3.0)	Atkinson, 2011	HIV+: 14%; HIV-: 6% (Lifetime alcohol use disorder, all were male)
		Jin, 2013	HIV+IDU: 15.7%; HIV-IDU: 19.3%; non-IDU: 12.4% (Lifetime alcohol use disorder)
	Questionnaire	Wu, 2006	10.2% (current use)
		Wu, 2007	8/175(4.6%, definition unknown)
		Fang, 2007	16.8% (definition unknown)
		Su, 2010	HIV+: 47.7%; HIV-: 54.9% (past month)
		Luo, 2013	Ever drinkers: 65.1% (male: 89.7%; female: 16.9%). Current drinker (past month): 40.0% (male: 35.9%; female: 7.1%).
		Dwyer, 2014	38% (past 6 months; male: 18%; female: 12.5%)
		Xu, 2014	79/157 (50.3%, past year)
		Sun, 2014	322/772 (41.7%, past month)
Tobacco use	Questionnaire	Wu, 2006	44.1% (current use)
		Wu, 2007	48/175 (27.4%, definition unknown)
		Fang, 2007	26.3% (definition unknown)
		Li, 2007	HIV+: 72.2%; HIV-: 40.4% (definition unknown)
		Su, 2010	HIV+: 41.2%; HIV-: 26.0% (past month)
		Cheng, 2014	15/68(22.1%, smoking history)
		Sun, 2014	373/772 (48.3%, "Are you a smoker?")
		Xu, 2014	75/157 (47.8%, past year)
		Dwyer, 2014	50% (Current smoker; male: 53.8%; female: 25.0%)

Notes: Questionnaire: not specified or self-developed instruments; CIDI: Composite International Diagnostic Interview

Other studies assessed substance use behavior mostly by self-developed questionnaires. In terms of drug use, three studies reported a range from 55.4% to 98.4% of lifetime drug use [44,109,110]. Greene et al. [37] found that among 96 HIV-positive people who used drugs recruited from a clinic in Yunnan, 16.7% reported injecting drugs daily. Wu et al. [41] found that 23.2% of newly diagnosed men who have sex with men in Chengdu were current heroin users. Finally, Fang et al. [39] conducted a survey among 572 pregnant women in four provinces who were diagnosed between 2004 and 2006 and found that 4.74% used drugs and most shared drug paraphernalia (74%).

As for alcohol use, three studies reported prevalence of alcohol use in the past month, which was from 40.0% to 47.7% [26,90,109]. In a cross-sectional study, 50.3% of HIV-positive men who have sex with men on ART had used alcohol in the last year [43]. After disaggregating findings by gender, males were more likely to report alcohol use than females [21,88,109]. In three case-control studies, people living with HIV reported comparable [26,56] or significantly higher alcohol use rates than control groups [21]. Three other studies did not specify the definition of substance use [28,67,70].

In terms of tobacco use, 41.2% of participants who were HIV-positive reported smoking in the past month, and their smoking frequency and quantity were both higher than the HIV-negative group in a community-based case-control study [26]. In a cross-sectional study, 47.8% of HIV-positive men who have sex with men on ART had smoked in the last year [43]. Several other studies reported smoking behaviors among people living with HIV without specifying definitions [28,42,67,70,88,90,93], and the smoking rates ranged from 22.1% to 50%. Only one study presented data separated by gender, and the researchers found that 53.8% of males and 25% of females were current smokers [88].

### The health and treatment-related correlates

#### Health-related correlates

The findings about the associations between mental health and physical health status, as measured by time since diagnosis, CD4 counts, number of somatic symptoms, and disease courses (AIDS diagnosis), are fragmented as well. Two studies found that people with a higher number of somatic symptoms were more likely to experience depression and anxiety [22,96], while two studies reported no significant association between them [24,91].

Meanwhile, eight studies showed that people living with HIV with lower CD4 (lower than 200) or in the AIDS stage had poorer mental health status [18,19,42,50–52,79,101], including depression, anxiety, suicidal ideation, and NP impairment, while another six studies reported that there was no association between CD4 count or being diagnosed with AIDS and mental health [19,21,58,67,89,91].

Studies also found that people living with HIV or in treatment for shorter periods were more likely to report poorer mental health status, including PTSD [105], suicidality [44], and self-reported depression [41,79] and anxiety [71]. Although it was a cross-sectional rather than longitudinal study, Ren et al. [44] found that the prevalence of suicide attempts increased along with disease progression. Yet, several studies reported depression [22,77,84,89,91], anxiety [22,91] and suicidal ideation [18,27] did not differ by time since diagnosis.

#### Treatment-related correlates

Similarly, there were inconsistent results about treatment-related correlates of mental health among people living with HIV in China. Two studies found that people on ART were more likely to report high levels of depression and anxiety [77,93]. One study indicated absence of ART was independently associated with higher depression score [24], whereas four studies suggested that mental health status (depression, anxiety, PTSD, and suicidal ideation) was not associated with ART treatment [18,42,89,105]. In addition, one study reported a negative association between adherence to ART and anxiety and depression [72], while one other study reported a non-significant connection [67].

## Discussion

In this review, the available evidence indicates that people living with HIV in China are at risk for mental health problems. Depression and anxiety were the most widely studied and the most common problems, while findings regarding suicidality, substance use, HAND, and PTSD were varied and less prevalent (S2 Table). Notably, most studies were based on a broad range of self-reported scales for psychiatric symptoms, and only three studies employed standardized clinical diagnostic measurements [21,38,44]. Although studies reported a high prevalence of psychiatric symptoms, the reported prevalence of psychiatric disorders was low. There was only around a 2% prevalence rate reported for both current major depressive disorder and substance use disorder [21,38]. Given health care strategies for psychiatric symptoms and psychiatric disorders may differ, we are not sure of the actual needs for different health services among this population. There is also not enough evidence to assist policy makers in making decisions about distribution of resources and cost-effectiveness.

We also found conflicting results regarding health and treatment related correlates of mental health, which may be due to the cross-sectional design of the majority of studies. In order to identify the relationship between disease severity and mental health status, a longitudinal design is needed (such as from HIV-infection diagnosis to AIDS diagnosis). Furthermore, the equivocal findings of our review may be because of the limitation in comparability and generalizability of the current studies. First, as mentioned above, the inconsistency in measurements limits the comparability across studies, especially on substance use and suicidality, of which the definitions were often not specified. Second, not all studies reported the sociodemographic and HIV-related characteristics of participants that may have a significant effect on mental health status. Third, most studies focused on subgroups with differential patterns, such as former blood/plasma donors [19–27], people who injected drugs [35,37,38], pregnant women [31,39,40], and men who have sex with men [41–43], rather than the wider population who are living with HIV in a particular region or the whole country.

According to our assessment criteria on sample size, sampling method, eligibility criteria, and participation rate, the results indicated the included studies were of low quality and had a high risk of bias. Convenience sampling was the most commonly used method, while almost one fourth did not specify the sampling strategy. Additionally, the majority of studies were based on a small sample size. As such, questions can be raised about sample representativeness and generalizability of the findings. Additionally, one-fourth of the studies employed a case-control design, but sometimes the HIV-negative status of the comparison group was self-reported or undetermined. Some studies also failed to present comparable sociodemographic characteristics with the HIV-positive group, which begs the question of whether there was an adequate control group. Further, many studies without a control group preferred to compare the results of people living with HIV with Chinese norms for the general population, especially those using the SCL-90. However, the most commonly used norm for the SCL-90 was based on a study conducted in 1986 [111], which may fail to serve as a good comparison because the psychological status of the general Chinese population may have greatly changed over the past three decades.

This review demonstrates the mental health risks for people living with HIV and reveals the need for greater support and prevention work. Reduction in the prevalence of mood disorders in people living with HIV should be a primary goal. Future studies should utilize larger and more rigorously characterized samples, as well as more sound methodologies. Research would also benefit from using consistent instruments for which data is already available in similar populations in the region. More studies are needed on suicidal behavior, substance use, HAND, and PTSD, as well as the co-occurrence of these problems, instead of just treating one problem as a risk factor for another. Additionally, more prospective longitudinal research is required to track the trend of mental health status concurrent with the natural history of disease.

It is important to note that, despite the obvious need for mental health services shown by this review, people living with HIV in China rarely access the support that they require [28]. Additionally, we found only one study that investigated mental health service utilization [27], and two studies indicated the need for psychological support for this population [22,24]. More research is also required to understand their mental health needs and develop effective intervention efforts to address this issue. Furthermore, although this review focused on quantitative data, qualitative evidence is also valuable. Because the qualitative reports are the voices of people living with HIV themselves—whether they view mental health as a problem and what they need in terms of care and support for mental health issues—a future review of qualitative evidence will aid greater learning and understanding of the mental health issues of this population.

## Conclusion

This review identified the vulnerability of people living with HIV in terms of mental health issues, and depression and anxiety are most prevalent. It is imperative for academics, health care providers, and policy makers to address this issue as a matter of urgency, and to involve people living with HIV as well as their caregivers in defining their psychiatric and psychological needs. Combined with qualitative evidence, large-scale and longitudinal studies using standard instruments are needed to better inform policies on, and services for, the complex and diverse needs of different subgroups of people with HIV. It is time to put mental health services for people living with HIV in China on the healthcare agenda and develop an integrated mental health and physical health service.

## Supporting Information

S1 File Search strategies. (DOCX)

S1 PRISMA Checklist Filled PRISMA Checklist. (DOCX)

S1 Table Overview of articles on the mental health of people living with HIV in China. (DOCX)

S2 Table Prevalence of mental health problems. (DOCX)
