# Supplementary material for: The Mental Health of People Living with HIV in China, 1998–2014: A Systematic Review
Source: PLoS One. 2016 Apr 15;11(4):e0153489. doi: 10.1371/journal.pone.0153489 (PMC4833336; doi:10.1371/journal.pone.0153489)
Supplement: S1 File — (DOCX) [file pone.0153489.s001.docx]

**Appendix S1. Search strategy for individual databases**

**Ovid Medline**

1. (mental$ adj2 (health or ill$ or disorder$ or disab$)).mp.

2. ((psychotic or mood or affective or stress or comment mental) adj2 disorder$).mp.

3. ((substance or drug$ or alcohol) adj3 (use$ or misuse or abus$)).mp.

4. (psychiatric or psychiatry or psychology$ or depress$ or anxiety$ or anxious or psychos$ or PTSD$ or suicide$ or neuropsycho$ or smok$).mp.

5. 1 or 2 or 3 or 4

6. exp HIV/

7. exp AIDS/

8. exp acquired immunodeficiency syndrome/

9. 6 or 7 or 8

10. 5 and 9

11. limit 10 to (english language and humans)

12. limit 11 to yr="1998-2014"

13. exp China/

14. 12 and 13

# Ovid PsycINFO

1. (mental$ adj2 (health or ill$ or disorder$ or disab$)).mp.

2. ((psychotic or mood or affective or stress or comment mental) adj2 disorder$).mp.

3. ((substance or drug$ or alcohol) adj3 (use$ or misuse or abus$)).mp.

4. (psychiatric or psychiatry or psychology$ or depress$ or anxiety$ or anxious or psychos$ or PTSD$ or suicide$ or neuropsycho& or smok$).mp.

5. 1 or 2 or 3 or 4

6. exp HIV/

7. exp AIDS/

8. exp acquired immunodeficiency syndrome

9. 6 or 7

10. 5 and 9

11. limit 10 to (english language and humans)

12. limit 11 to yr="1998-2014"

13. exp China

14. 12 and 13

**Web of science-core collection**

#1

TS=(mental and (health or ill* or disorder* or disab*))

*DocType=Article; Language=English; Time range=1998-2014*

#2

TS=((psychotic or mood or affective or stress or comment mental) and disorder*)

*DocType=Article; Language=English; Time range=1998-2014*

#3

TS=((substance or drug* or alcohol) and (use* or misuse or abus*))

*DocType=Article; Language=English; Time range=1998-2014*

#4

TS=(psychiatric or psychiatry or psychology* or depress* or anxiety* or anxious or psychos* or PTSD* or suicide* or neuropsycho* or smok*)

*DocType=Article; Language=English; Time range=1998-2014*

#5= #1 OR #2 OR #3 OR #4

#6

TS=(HIV OR AIDS OR acquired immunodeficiency syndrome)

*DocType=Article; Language=English; Time range=1998-2014*

#7= #5 AND #6

#8

CU=(China) or PS=(china or chinese or beijing or shanghai or tianjin or hebei or shanxi or "inner mongolia" or liaoning or jilin or heilongjiang or jiangsu or zhejiang or anhui or fujian or jiangxi or shandong or henan or hunan or guangdong or guangxi or hainan or chongqing or sichuan or guizhou or yunnan or tibet or shaanxi or gansu or qinghai or ningxia or xinjiang or hongkong)

*DocType=Article; Language=English; Time range=1998-2014*

#9= #7 AND #8

**Pubmed**

1.

((((mental and (health or ill* or disorder* or disab*))) OR ((psychotic or mood or affective or stress or comment mental) and disorder*)) OR ((substance or drug* or alcohol) and (use* or misuse or abus*))) OR (psychiatric or psychiatry or psychology* or depress* or anxiety* or anxious or psychos* or PTSD* or suicide* or neuropsycho* or smok*)

[All field]

2.

((HIV) OR AIDS) OR acquired immunodeficiency syndrome

[All field]

3.

(china or chinese or beijing or shanghai or tianjin or hebei or shanxi or "inner mongolia" or liaoning or jilin or heilongjiang or jiangsu or zhejiang or anhui or fujian or jiangxi or shandong or henan or hunan or guangdong or guangxi or hainan or chongqing or sichuan or guizhou or yunnan or tibet or shaanxi or gansu or qinghai or ningxia or xinjiang or hongkong)

[All field]

4. 1 AND 2 AND 3

5. limit 4 to (english language and humans)

6. limit 5 to yr="1998-2014"

# References in Chinese databases

1. **China National Knowledge Infrastructure (CNKI,中国知网)：** <http://epub.cnki.net/grid2008/index/ZKCALD.htm>

**Search query**

|  | Keywords in Chinese | English translation |
| --- | --- | --- |
| 1 | Exp 精神卫生 | Exp mental health |
| 2 | Exp 心理卫生 | Exp psychological health |
| 3 | Exp 心理问题 | Exp psychological problem |
| 4 | Exp 情绪问题 | Exp emotional problem |
| 5 | EXP物质滥用 | EXP substance abuse |
| 6 | EXP酒精使用 | EXP alcohol use |
| 7 | EXP焦虑 | EXP anxiety |
| 8 | EXP抑郁 | EXP depression |
| 9 | EXP自杀 | EXP suicide |
| 10 | EXP 应激障碍 | EXP stress related disorder |
| 11 | EXP 神经心理 | EXP neuropsychology |
| 12 | OR between (1 to 11, exact match) | |
| 13 | EXP艾滋病 | EXP acquired immunodeficiency syndrome |
| 14 | EXPAIDS | EXP AIDS |
| 15 | EXP HIV | EXP HIV |
| 16 | OR between(12 to 14, exact match) | |
| 17 | 1998-01-01——2014-12-31 | |
| 18 | 12 and 16 and 17 | |
| 19 | Literature types include journal articles, conference proceedings, thesis, year book. (期刊论文 + 会议论文 + 学位论文 +年鉴) | |

Exp: Expansions in Chinese and English

1. **CBM: (China BioMedical literature Database, 中国生物医学文献数据库):** <http://www.sinomed.ac.cn/zh/>

("艾滋病"[全字段:智能] OR "HIV"[全字段:智能] OR "AIDS"[全字段:智能]) AND ("精神卫生"[全字段:智能] OR "心理卫生"[全字段:智能] OR "心理问题"[全字段:智能] OR "情绪问题"[全字段:智能] OR "物质滥用"[全字段:智能] OR "酒精使用"[全字段:智能] OR "焦虑"[全字段:智能] OR "抑郁"[全字段:智能] OR "自杀"[全字段:智能] OR "神经心理"[全字段:智能] OR "应激障碍"[全字段:智能] OR "吸烟"[全字段:智能])

Limited to yr="1998-2014"

1. **Digital journal of Wanfang Data (ChinaInfo, 万方数据资源系统)** ：<http://librarian.wanfangdata.com.cn/default.aspx?dbid=Paper>

(主题:(抑郁) + (精神卫生) + (心理卫生) + (情绪问题) + (物质滥用) + (酒精使用) + (焦虑) + (自杀) or (神经心理) + (应激障碍)+(吸烟))* （主题:(艾滋病) + (HIV) +(AIDS)） * Date:1998-2014
