# Supplementary material for: The Mental Health of People Living with HIV in China, 1998–2014: A Systematic Review
Source: PLoS One. 2016 Apr 15;11(4):e0153489. doi: 10.1371/journal.pone.0153489 (PMC4833336; doi:10.1371/journal.pone.0153489)
Supplement: S1 Table — (DOCX) [file pone.0153489.s003.docx]

**S1 Table Overview of articles on the mental health of PLWHA in China**

| **First author, year** | **Study site; setting** | **Study design** | **Sampling** | **Sample** | **Instruments** | **Results** | **Risk of bias** |
| --- | --- | --- | --- | --- | --- | --- | --- |
| Li, 2004 | Sichuan; in CDC registry | Case-control | N/A | ① 71 HIV+ adults; 9.9% female; age 28.4±6.7 years. ② 162 HIV+ patients’ relatives; 56.2% female; age 43.8±16.3 years. ③ 97 HIV- controls. | SDS, SAS, self-develop instrument | Depression, anxiety, substance abuse | 0 |
| Chen, 2004 | Yunnan; hospital | Case-control | N/A | ① 89 HIV+ adults; 33.7% female; age 32±9 years; time since diagnosis ranged from 3 months to 6 years. ② 89 demographic-comparable local HIV- controls | SCL-90 | Psychological distress | 1 |
| Huang, 2004 | Yunnan; hospital | Cross-sectional | N/A | 19 HIV+ adults；48.4% female; age 30.37±5.96 years; 63.1% AIDS. | SCL-90 | Psychological distress | 1 |
| Qu, 2005 | Beijing; hospital | Retrospective study | Complete sample | 848 HIV+ patients of this hospital. | Medical records | Completed suicide, suicide behavior | 1 |
| Zhang, 2005 | Hubei; in CDC registry | Cross-sectional | N/A | 162 rural HIV+ adults; 44.4% female; age 40.24±2.78 years; 58.6% AIDS. | SCL-90, HAMA, HAMD | Psychological distress, depression, anxiety | 0 |
| Wu, 2006 | Liaoning & Jilin; in CDC registry | Cross-sectional | Complete sample | 59 HIV+ adults on ART more than 3 month; 47.5% female; age 44.5±7.5 years; 38.9% CD4<200. | SAS, SDS, self-develop instrument | Depression, anxiety, alcohol use, tobacco use | 3 |
| Qian, 2006 | Sichuan & Hubei; N/A | Cross-sectional | Convenience | 98 HIV+ adults; 22.45% female; age 35.22±1.72 years. | SDS, SAS | Depression, anxiety | 1 |
| Chan, 2006 | Hong Kong; hospital clinic | Cross-sectional | N/A | 118 HIV+ patients | HADS | Anxiety, depression | 1 |
| Sun, 2007 | Central China; health care center | Cross-sectional | Purposive sampling | 94 HIV+ adults; 44.6% female; mean age 42.9 years; 51.1% on ART | SCL-90 | Psychological distress | 2 |
| Lv, 2007 | A county in central China; community sample | Cross-sectional | Random | 269 rural HIV+ adults who have history of paid blood donation; 53.5% female; 41.7% CD4<200 | DASS, self-develop instrument | Depression, anxiety, suicide behavior | 3 |
| Wu H, 2007 | An'hui; community sample | Cross-sectional | Convenience | 175 rural AIDS patients; 56% female; age 44.26±8.36 years | SDS, self-develop instrument | Depression, suicide behavior, alcohol use, tobacco use | 0 |
| Fang, 2007 | An'hui; N/A | Cross-sectional | N/A | 95 rural HIV+ adults who have history of paid blood donation; 57.9% female; age 45.19±7.71 years | SAS, SDS, self-develop instrument | Depression, anxiety, alcohol use, tobacco use | 0 |
| Li, 2007 | Xinjiang, Shanxi, Henan; N/A | Case-control | Convenience | ① 393 HIV+ adults; 20.9% female; age 18-71 years; 14.2% AIDS；36.1% on ART; 95.7% time since diagnosis ≤3 years.  ② 94 age-comparable HIV- controls; 45.7% female; age 20-68 years. | HADS, self-developed instrument | Anxiety/depression, tobacco use | 2 |
| Hu, 2007 | Zhejiang; in CDC registry | Case-control | Random | ①214 HIV+ adults; 24.3% female; age 31.32±9.15 years. ② 150 demographic-comparable HIV- controls; 33.3% female; age 30.45±8.23 years. | SCL-90 | Psychological distress | 1 |
| Pei, 2007 | Guangdong; hospital | Case-control | N/A | ① 26 HIV+ with TB; 11.5% female; age 20-45 years. ② 40 HIV- with TB; 45% female; age 20-59 years. | SCL-90, SDS | Psychological distress, depression | 0 |
| Chen, 2007 | Hubei; N/A | Cross-sectional | Random | 105 rural HIV+ patients; 43.3% female; age 8-64 years | SAS, SDS | Anxiety, depression | 1 |
| Wu Y, 2007 | Shanghai; hospital | Prospective study | Complete sample | 36 AIDS managed by hospital between 1991.1-2003.12 | Medical records | AIDS dementia complex | 0 |
| Shi, 2008 | Shanxi; community sample | Case-control | Random | ① 81 rural HIV+ adults; 40.7% female. ② 64 PLWHA’s relatives; 57.8% female. ③ 46 HIV- controls; 60.9% female. | SAS, SDS, | Anxiety, depression | 2 |
| Li B, 2008 | Yunnan; hospital | Cross-sectional | Convenience | 95 HIV+ adults; 38.9% female; age 36.2±6.46 years; 68.55% time since diagnosis in 1 year. | SAS, SDS | Anxiety, depression | 1 |
| Li K, 2008 | An'hui; community sample | Cross-sectional | Systematic | 60 rural HIV+ adults infected by paid-blood-donation; 61.7% female; age 45.98±8.20 years. | SCL-90 | Psychological distress | 0 |
| Bo，2008 | Shanxi; hospital | Case-control | N/A | ① 56 HIV+ adults; 57.1% female; age 38±12 year. ② 30 HIV- controls; 53.3% female; age 44±16 years. | SAS | Anxiety | 1 |
| Wang， 2008 | Hunan; clinics forART | Cross-sectional | Convenience | 111 HIV+ adults infected by injected drug use; all on ART more than 1 month; 16.2% female age 36.7±6.7 years; time since diagnosis 1.4±0.96 years; 49.5% CD4<200. | SDS | Depression | 1 |
| Chen G, 2008 | Henan; in CDC registry | Cross-sectional | Complete sample | 192 rural HIV+ adults; 52.1% female; 75.5% AIDS; age 43.94±9.07 years. | SCL-90 | Psychological distress | 2 |
| Chen X, 2008 | Guangxi; hospital | Cross-sectional | Convenience | 82 HIV+ inpatients; 25.6% female; 30.5% AIDS; age 34.43±9.63. | SDS | Depression | 2 |
| Wright，2008 | Beijing & Hong Kong; hospital | Cross-sectional | N/A | 50 HIV+ outpatient in Beijing; 62 HIV+ outpatient in Hong Kong | Neuropsychological test battery, CES-D | Neurocognitive impairment, depression | 2 |
| Heaton, 2008; Atkins, 2011 | An'hui; community sample | Case-control | Convenience | ①203 HIV+ rural former plasma donors (FPD); 39% female; age 40.2±6.4; 56% AIDS. ②198 HIV-rural FPD; 39% female; age 40.3±6.3 years. | CIDI (3.0), BDI, NP test battery | MDD, alcohol use disorder, other substance disorder, suicide, NP impairment | 2, 1 |
| Au, 2008 | Hong Kong; hospital clinic | Cross-sectional | N/A | 90 HIV+ adults on ART; 17.8% female; age 39.24±6.51 years; 84.5% time since diagnosis >2 years; mean CD4 count 386.86±222.605 | STAI, BDI；Hong Kong List learning Test (HKLLT); | Anxiety, depression, memory deficits | 1 |
| Fang, 2008 | Henan, Guangxi, Yunnan, Xinjiang; N/A | Cross-sectional | Consecutive | 572 HIV+ women who got diagnosis during pregnancy between 2004 to 2006; median age 27 years. | N/A | Drug use | 1 |
| Shan, 2009 | Guangdong; N/A | Cross-sectional | Consecutive | 250 newly diagnosis HIV+ adults between Jan to Dec 2007; all male; mean age 32.7 years. | N/A | Drug use | 0 |
| Yu, 2009 | A rural county in central China; community sample | Case-control | Random sampling and convenience sampling | ① 271 rural HIV+ adults who were former plasma/blood donors; 53.1% female; 81.7% on ART; 70.7% time since diagnosis in 3-5 years. ② 67 HIV- villagers; 79.1% female. | DASS-21 | Depression, anxiety | 3 |
| Ren, 2009 | Guangdong; in CDC registry | Cross-sectional | Convenience | 342 HIV+ adults; 14.8% female; 17.8% AIDS; age 34.5±7.1 year. | SCID-I/P, self-develop instruments | MDD, GAD, suicide behavior, drug use | 3 |
| Lu, 2009 | Guangzhou; hospital | Cross-sectional | N/A | 102 HIV+ adults; 33.3% female; 60.8% CD4<200 | SCL-90, SDS, SAS | Psychological distress, depression, anxiety | 1 |
| Yin, 2009 | Hubei; hospital | Case-control | N/A | ① 102 HIV+ adults; 29.4% female; age 44.25±8.35 years; 52.9% AIDS.  ② 102 HIV- and demographic-comparable controls recruited from a clinic. | SCL-90 | Psychological distress | 1 |
| Bao, 2009 | Shanghai; clinics | Cross-sectional | Convenience | 80 HIV+ adults; 36.3% female; age 21-58 years. | SCL-90 | Psychological distress | 1 |
| Liu, 2010 | Shandong; in CDC registry | Cross-sectional | Complete sample | 624 HIV+ patients; 45.4% female; age 34.37±11.38; non-AIDS patients; all CD4≥200 | SDS, SAS | Depression, anxiety | 2 |
| Guli, 2010 | Xinjiang; hospital | Case-control | N/A | ① 97 AIDS adults； 37.1% female; age 32.41±7.8 years.  ② 100 HIV- outpatients for medical examination | SCL-90 | Psychological distress | 0 |
| Shi, 2010 | Hunan; N/A | Case-control | Cluster random sampling | ① 51 HIV+ adults; 25.5% female; age 49±15 year.  ② 49 relatives of PLWHA; 63.3% female; age 38±8.  ③ 96 controls; 44.8% female; age 36±9 years. | IDA | Depression, anxiety | 1 |
| Pan, 2010 | Shanghai; clinics | Cross-sectional | Convenience | 254 AIDS adults; 9.8% female; age 40.4±11.4 years. | SAS | Anxiety | 2 |
| Wang J, 2010 | Beijing; hospital | Case-control | N/A | ① 94 HIV+ adults; 14.9% female; age 22-51 years.  ② 38 employees of the hospital; 10.5% female; age 38.3±10.7 years. | SCL-90 | Psychological distress | 0 |
| Wang, Y, 2010 | Yunnan; community sample | Cross-sectional | Random | 44 rural HIV+ pregnant woman; mean age 25.7 years | SDS, SAS | Depression, anxiety | 1 |
| Su, 2010 | An'hui; community sample | Case-control | N/A | ① 153 rural HIV+ married people infected by paid-blood-donation; 56.2% female; age 40.38±6.19 years.  ② 153 age- and gender-comparable married HIV- adults; 56.2% female; age 39. 88 ± 7. 21 years. | SCL-90, unhealthy behavior questionnaire for adults | Psychological distress, suicide behavior, alcohol use, tobacco use. | 0 |
| Gao, 2010 | Guangzhou; CDC, hospital and AIDS monitoring setting | Cross-sectional | Convenience | 250 HIV+ patients; 34.4% female; age 36.04±8.16 years; 72% CD4<200. | SAS, CES-D | Anxiety, depression | 1 |
| Lu, 2010 | Henan; N/A | Cross-sectional | N/A | 144 rural AIDS on ART; 36.1% female; age 18-73 years; time since diagnosis 3-17 years. | SDS | Depression | 1 |
| Huang, 2010 | Henan; in CDC registry | Case-control | N/A | ① 59 HIV+ adults; 40.7% female; age 28.2±8.9 years.  ② 59 HIV- controls; 38.9% female. | SDS, SAS | Depression, anxiety | 1 |
| Jin, 2010 | Zhejiang; CDC | Case-control | N/A | ①214 HIV+ adults; 24.3% female; age 31.32±9.15 years; all CD4 >350, no clinical symptoms of AIDS, and not on ART. ② 200 HIV- adults; 25.0% female; age 30.45±8.23 years. | SCL-90 | Psychological distress | 1 |
| Lau, 2010 | A rural county in central China; community sample | Cross-sectional | Random | 176 HIV+ adults who were former blood and/or plasma donors (FBPD) and married; 54.0% female; 88.6% on ART; 88% time since diagnosis over 2 years. | DASS, self-developed instrument | Depression, anxiety, suicide behavior | 2 |
| Meade, 2010 | Henan; community sample | Cross-sectional | Convenience | 207 rural HIV+ FBPD; 54% female; age 39.6±7.0 years; time since diagnosis 2.9±1.6 years | BDI, SAS | Depression, anxiety | 2 |
| Cysique, 2010 | An'hui; community sample | Cohort | Complete sample and random sampling | ①Baseline (Heaton, 2008; Atkins, 2011) ② 1-year follow up, all available HIV+ FPD (N=192) were reassessed, 5.4% attrition rate. ③ 101 HIV- controls were randomly selected and reassessed. | NP test battery, BDI | Neuropsychological impairment, depression | 1 |
| Li, 2011 | An'hui; community sample | Cross-sectional | Convenience | ① 79 rural HIV+ adults; 36.7% female. ② 79 HIV- family members | SDS | Depression | 2 |
| Liu, 2011 | Hubei; clinics | Cross-sectional | Judgment sampling | 106 HIV+ adults; 50.9% female; age 32.64±10.78 years; mean time since diagnosis 1.5 years; 67% on ART. | SDS | Depression | 2 |
| Wu, 2011 | Guangxi; in CDC registry | Cross-sectional | N/A | 54 HIV+ adults; 40.7% female; age 40.24±2.78 years; 16.7% AIDS | SCL-90, SDS, SAS | Psychological distress, depression, anxiety | 0 |
| Sun, 2011 | Shandong; N/A | Cross-sectional | Convenience | 36 HIV+ patients; 41.7% female; mean age 35 years; 33.3% on ART. | SAS, SDS | Anxiety, depression | 0 |
| Yang, 2011 | Yunnan; N/A | Case-control | Consecutive and convenience sampling | ① 307 HIV+ pregnant women; age 28.3±5.3 years.  ② 160 HIV- pregnant women; age 26.2±4.2 years. | SCL-90 | Psychological distress | 3 |
| Hu, 2011 | Henan; in CDC registry | Cross-sectional | Cluster | 612 rural PLWHA on ART who have history of paid-blood-donation.53.3% female; age 45.2±8.7 years. | SCL-90 | Psychological distress | 3 |
| Dong, 2011 | Guangxi; clinics | Cross-sectional | Random | 400 HIV+ adults; 36.25% female; age 39.26±10.99 years; 79% AIDS. | SDS | Depression | 2 |
| Lai, 2011 | Sichuan; in national DataFax ART registry | Retrospective study | Complete sample | 766 HIV+ adults who started ART before Sep. 30^th^, 2008; 26.5% female; age 37.14±9.68 years. | Death records | Completed suicide | 2 |
| Zhen, 2011 | Henan; in clinic registry | Cross-sectional | Convenience | 160 AIDS patents who were on ART; 35.0% female. | SAS | Anxiety | 1 |
| Lu, 2012 | Kunming; hospital | Case-control | Complete sample | ① 18 HIV+ newly diagnosis inpatients; 44.4% female; age 51.09±4.58 years. ② 72 HIV- inpatients; 47.2% female; age 50.31±5.64 years. | SCL-90 | Psychological distress | 2 |
| Yang, 2012 | Guangzhou; in CDC VCT clinics | Cross-sectional | N/A | 144 HIV+ adults; 67.38% female; age 36.9±10.8 years; 31.3% AIDS; mean time since diagnosis 1.84±1.68 years; 15.97% CD4<200. | SAS | Anxiety | 2 |
| Bai, 2012 | Hunan; in CDC clinic | Cross-sectional | N/A | 145 HIV+ adults; 43.4% female. | BDI | Depression | 0 |
| Wang, 2012 | Yunnan; hospital | Cross-sectional | Complete sample | 38 HIV+ inpatients; 39.5% female; age 31±2.21 years. | SCL-90 | Psychological distress | 2 |
| Yuan, 2012 | Henan; in Chi Heng Foundation (CHF) registry | Cross-sectional | Random | 201 rural HIV+ adults; 50.2% female; age 30-60 years. | BDI | Depression | 2 |
| Xie S, 2012 | Henan; hospital and community sample | Cohort | N/A | 106 HIV+ adults; 41.5% female; age 45.69±8.50 years; 56.60% time since diagnosis over 10 years. | SCL-90 | Psychological distress | 2 |
| Xie Z, 2012 | Henan; community sample | Cross-sectional | Random | 1064 rural HIV+ adults; 54.9% female; age 40.54±5.34 years. | SCL-90 | Psychological distress | 2 |
| Zhao, 2012 | Yunnan; clinics | Cross-sectional | N/A | 32 HIV+ adults; 31.3% female; mean age 32.3 years. 56.3% in asymptomatic period. | SCL-90 | Psychological distress | 1 |
| Zhang, 2012 | Henan, Yunnan, Beijing; hospital | Cross-sectional | N/A | 134 HIV+ adults; 43.3% female | IHDS and neuropsychological test battery. | HAND | 2 |
| Rao, 2012 | Beijing； hospital | Cross-sectional | N/A | 120 HIV+ adults; 18% female; age 36±8.0 years; 58% CD4<200 | CES-D | Deprssion | 1 |
| Greene, 2013 | Yunnan; clinic | Cross-sectional | Convenience | 96 HIV+ injection drug users; 39.6% female; age 41.1±4.9 years | HADS, self-develop instruments | Depression, drug use | 2 |
| Jin, 2013 | Yunnan; clinics | Case-control | Convenience | ① 204 HIV+ heroin IDUs in methadone treatment; 34.2% female; age 36.8±4.9 years; 28.4% AIDS; 24.0% on ART. ② 202 HIV- heroin IDUs in methadone treatment; 34.8% female; age 34.5±4.4 years. ③201 non-IDU controls; 34.3% female; age 34.9±6.4 years. | CIDI (3.0), BDI-2, | MDD, depression, alcohol use disorder, heroin use disorder, suicide behavior | 1 |
| Luo, 2013 | Yunnan; in CDC registry | Cross-sectional | Convenience | 455 rural HIV+ adults; 33.8% female; age 38.1±8.8 years; 67% on ART. | Self-develop instrument | Alcohol use and drug use | 3 |
| Su, 2013 | Hunan and Guangdong; clinics and CDC | Cross-sectional | Convenience | 258 HIV+ adults; 26.4% female; 35.4% time since diagnosis over 2 years; 43.8% on ART | BDI-2 | Depression | 1 |
| Wu, 2013 | Liaoning; CDC | Cross-sectional | Random | 424 HIV+ employees; 9.0% female; age 39.30±11.59 years. | SAS | Anxiety | 3 |
| Li, 2013 | Hunan; CDC | Cross-sectional | Convenience | 264 HIV+ adults; 33.7% female; age 40.96±13.28 years; 35.6% AIDS; 66.3% on ART. | PCL | PTSD | 2 |
| Yang, 2013; Qin, 2014 | Guangdong; CDC VCT clinics | Cross-sectional | N/A | 144 HIV+ adults; 32.64% female; age 36.9±10.8 years; 31.3% AID; time since diagnosis 1.84±1.68 years. | SDS, SIOSS | Depression, suicide ideation | 1, 1 |
| Guo, 2013 | N/A | Cross-sectional | N/A | 35 HIV+ adults with 85.7% rural; 42.9% female; mean age 42.8 years. | SCL-90, SDS, SAS | Psychological distress, depression, anxiety | 0 |
| Huang, 2013 | Guangxi; hospital | Cross-sectional | Random | 102 HIV+ patients; 16.7% female; mean age 35 years. | SAS, SDS | Anxiety, depression | 2 |
| Zhao, 2013 | Guangxi; hospital | Case-control | Multiple-stage stratified random sampling | ① 230 HIV+ inpatient; 33.9% female; age 47.8±10.6 years. ② 99 HIV- controls; 41.4% female; age 48.2±9.9 years. | IHDS | HAND | 1 |
| Zhen, 2013 | Shanghai; clinic | Cross-sectional | N/A | 136 HIV+ adults; 13.2% female; age 37±11 years; 69.1% on ART. | Montreal cognitive assessment (MoCA) | Neurocognitive impairment | 1 |
| Liu, 2014 | Hunan; in CDC registry | Cross-sectional | Consecutive | 290 HIV+ adults; 20.3% female; age 32.8±9.5 years. | PHQ-9, GAD-7 | Depression, anxiety | 1 |
| Zhou Z, 2014 | Jiangsu; hospital | Cross-sectional | N/A | 41 HIV+ adults; 19.5% female; mean age 43.19 years; 48.8% time since diagnosis < 1 year. | SCL-90 | Psychological distress | 0 |
| Zhou G, 2014 | Yunnan; hospital | Cross-sectional | Convenience | 356 HIV+ adults on ART; 43% female; .age 38.05±8.34 years; 25.8% CD4<200. | HADS | Anxiety | 3 |
| Yao, 2014 | Shanghai; clinics | Cross-sectional | N/A | 136 HIV+ adults; 3.7% female; mean age 37 years; mean time since diagnosis 1 year. | SCL-90, SDS, SAS | Psychological distress, depression, anxiety | 1 |
| Cheng, 2014 | Jiangxi; in CDC registry | Cross-sectional | Convenience | 68 HIV+ MSM; 36.8% CD4≤200. | SAS，SDS | Anxiety, depression, tobacco use | 2 |
| Qiu, 2014 | Hunan; in CDC registry | Cross-sectional | Consecutive | 370 newly identified HIV+ patients; 7.8% female; age 33±11 years; 32.7% in symptom phase | PHQ-9, GAD-7 | Depression, anxiety | 3 |
| Zou, 2014 | Yunnan; N/A | Cross-sectional | Random | 103 HIV+ patients with TB; 41.7% female; mean age 35 years. | SDS, SAS | Depression, anxiety | 1 |
| Chen, 2014 | Guangxi; in hospital clinics | Case-control | N/A | ① 142 HIV+ adults; 40.1% female; mean age 41.75 years; 46.5% time since diagnosis over 3 years. ② 129 demographic-comparable HIV- controls | HAMA, HAMD | Anxiety, depression | 0 |
| Gao, 2014 | Guangxi; N/A | Case-control | N/A | ①103 HIV+ adults with TB; 31.1% female; mean age 45.3 year. ② 103 HIV- controls; 31.1% female; mean age 44.8 years. | SCL-90 | Psychological distress | 2 |
| Xu, 2014 | Jiangsu; CDC | Cross-sectional | Cluster | 157 HIV+ MSM on ART; median age 40 years. | Self-developed instrument | Alcohol use and tobacco use | 2 |
| Dwyer, 2014 | Beijing; hospital | Cross-sectional | Convenience | 50 HIV+ adults; 16% female; age 35±7.3 years; 68.0% on ART. | CES-D, neuropsychological test, self-developed instrument | Depression, neuropsychological impairment, alcohol use, tobacco use | 2 |
| Wang, 2014 | Hunan; clinics | Cross-sectional | Convenience | 496 HIV+ adults; 31.0% female; mean age 38 year; 57.1% time since diagnosis in 2 years; 45.0% CD4<200; 74.3% on ART. | CES-D, self-developed instrument | Depression, drug use | 3 |
| Wu, 2014 | Chengdu; N/A | Cross-sectional | Convenience | 225 newly diagnosis HIV+ MSM in 1 year; 20.44% on ART | DASS, self-develop instruments | Depression, suicide behavior | 1 |
| Sun, 2014 | Liaoning; CDC | Cross-sectional | Random | 772 HIV+ adults; 10.5% female; age 37.4±11.2 years; 32.5% AIDS; 35.2% on ART. | SAS, CES-D, self-developed instrument | Anxiety, depression, alcohol use, tobacco use | 4 |

Notes:

N/A=not applicable; SDS= Zung Self-Rating Depression Scale; SAS= Zung Self-Rating Anxiety Scale; SCL-90=Symptom Checklist-90; HAMA= Hamilton Anxiety Scale; HAMD= Hamilton Depression Scale; HADS= Hospital Anxiety and Depression Scale; DASS= Depression Anxiety Stress Scale; CIDI= Composite International Diagnostic Interview; BDI= Beck Depression Inventory; STAI= State-Trait Anxiety Inventory; SCID-I/P= Structured Clinical Interview for DSM-IV; IDA= Irritability, Depression and Anxiety Scale; CES-D= The Centers for Epidemiological Studies Depression Scale; IHDS= International HIV Dementia Scale; PCL= PTSD Checklist; SIOSS= Self-Rating Idea of Suicide Scale; PHQ-9= Patient Health Questionnaire; GAD-7= Generalized Anxiety Disorder Scale; MDD= Major Depression Disorder; GAD= Generalized Anxiety Disorder
