# Supplementary material for: The Mental Health of People Living with HIV in China, 1998–2014: A Systematic Review
Source: PLoS One. 2016 Apr 15;11(4):e0153489. doi: 10.1371/journal.pone.0153489 (PMC4833336; doi:10.1371/journal.pone.0153489)
Supplement: S2 Table — (DOCX) [file pone.0153489.s004.docx]

**Table S2 Prevalence of mental health problems**

| **Mental health problems** | **Prevalence** |
| --- | --- |
| **Depression** |  |
| Lifetime major depressive disorder | 6.4% [38]-22.8 [44] |
| Current major depressive disorder | 1.0% [38]-2.0% [21] |
| Depressive symptoms | 16% [85]-100% [83], median 60.64% |
| **Anxiety** |  |
| Lifetime general anxiety disorder | 15.8% [44] |
| Anxiety symptoms | 11.11% [78]-97.53% [47], median 43.13% |
| **Suicidal behavior** |  |
| Completed suicide | 2.1% [104]-2.2% [103] |
| Suicide attempts | Lifetime: 2% [21]-37.7% [44] |
|  | Past 1 year: 5.9% [22] - 29.5%[44] |
|  | Past 6 months: 0.7% [26] |
|  | Since HIV diagnosis: 2.67% [41] |
| Suicide ideation | Lifetime: 13.7% [44] -34.8%[28] |
|  | Past 1 year: 3.8% [44] -34.1% [27] |
|  | Past 6 months: 5.9% [26] |
|  | Past 2 weeks: 14% [21] -37.1% [28] |
|  | Since HIV diagnosis: 48% [41] |
| Suicide plan | Lifetime: 8% [44]-14.0%[28] |
|  | Past 6 months: 2.6% [26] |
| **Posttraumatic stress disorder** | 46.2% [105] |
| **HIV-associated neurocognitive disorders** | 4% [85] -69.4% [88] |
| **Substance use** |  |
| Drug use | Current heroin use: 1.0% [38]-23.2%[89] |
|  | Lifetime: 55.4% [109] -98.4% [110] |
|  | Daily: 16.7% [37] |
| Alcohol use | Lifetime alcohol use disorder: 14% [21]-15.7% [38] |
|  | Current: 10.2% [67] - 41.7% [90] |
| Tobacco use | Current: 41.2%[26]-50%[88] |
